# Evaluation of enamel thickness of mandibular incisors

**DOI:** 10.1590/2177-6709.28.2.e2321149.oar

**Published:** 2023-05-29

**Authors:** Elisavet KONSTANTINIDOU, Eustaquio ARAUJO, Julie MCCRAY, Hiroshi UENO, Patricia Pigatto SCHNEIDER, Patrick Francis FOLEY

**Affiliations:** 1 Saint Louis University, Center for Advanced Dental Education (Saint Louis, Missouri, USA).; 2 Universidade Estadual Paulista, Faculdade de Odontologia (Araraquara/SP, Brazil).

**Keywords:** Enamel thickness, Mandibular incisors

## Abstract

**Objective:**

To measure enamel thickness at the proximal surfaces of the mandibular incisors, using micro-computed tomography (micro-CT) scans.

**Material and Methods:**

Forty-one single-rooted mandibular incisors were selected and analyzed according to anatomical characteristics, to form three groups: Group 1 - central incisors (n = 18); Group 2 - right lateral incisors (n = 10); and Group 3 - left lateral incisors (n = 13). First, enamel thickness at the proximal contact areas of the mandibular incisors was measured. Second, the mesial and distal surfaces of the lateral incisors were compared. Finally, the relationship between the tooth width and the mean enamel thickness was determined. Each tooth was scanned with a micro-CT scanner, and the image was processed with SCANCO micro-CT onboard analysis software.

**Results:**

There were no statistically significant differences in mean enamel thickness between the mesial and distal surfaces for each lateral incisor, or between contralateral lateral incisors. In all surfaces analyzed, the upper zones had statistically significantly thinner enamel (0.52 ± 0.10 mm) when compared to the middle and lower zones (0.60 ± 0.08 mm and 0.59 ± 0.08 mm, respectively). There was no correlation (r =0.07) between enamel thickness of the mandibular incisor and the tooth width.

**Conclusions:**

The enamel thickness of the mandibular incisors is similar on the mesial and distal surfaces, with the thinnest layer located at the upper zone.

## RESUMO

**Objetivo:**

Medir a espessura do esmalte nas superfícies proximais dos incisivos inferiores, usando imagens de microtomografia computadorizada (micro-CT).

**Material e Métodos:**

Quarenta e um incisivos inferiores com raiz única foram selecionados e analisados de acordo com as características anatômicas, formando três grupos: Grupo 1 - incisivos centrais (n = 18); Grupo 2 - incisivos laterais direitos (n = 10); e Grupo 3 - incisivos laterais esquerdos (n = 13). Primeiro, foi medida a espessura do esmalte nas áreas de contato proximal dos incisivos inferiores. Em segundo lugar, as faces mesial e distal dos incisivos laterais foram comparadas. Por fim, foi determinada a relação entre a largura do dente e a espessura média do esmalte. Cada dente foi escaneado com um *scanner* micro-CT, e a imagem foi processada com o *software* de análise SCANCO micro-CT.

**Resultados:**

Não houve diferenças estatisticamente significativas na espessura média do esmalte entre as superfícies mesial e distal de cada incisivo lateral, ou entre os incisivos laterais contralaterais. Em todas as superfícies analisadas, as zonas superiores apresentaram esmalte significativamente mais fino (0,52 ± 0,10 mm) quando comparadas às zonas média e inferior (0,60 ± 0,08 mm e 0,59 ± 0,08 mm, respectivamente). Não houve correlação (r = 0,07) entre a espessura do esmalte do incisivo inferior e a largura do dente.

**Conclusões:**

A espessura do esmalte dos incisivos inferiores é semelhante nas faces mesial e distal, com a camada mais fina localizada na zona superior.

## INTRODUCTION

Ballard introduced interproximal enamel reduction (IPR) of the mandibular incisors in 1944 as an orthodontic method to correct tooth discrepancy and obtain the necessary space for the dental alignment.^1^ At present, the popular instruments for IPR include abrasive metal strips, diamond-coated stripping disks, and air-rotor stripping devices.^2^

The main indications for IPR include mild to moderate crowding, a Bolton discrepancy, enhancement of tooth shape and dental esthetics, improvement of retention and stability following orthodontic treatment, normalization of gingival outline, and removal of black gingival triangles.^2^ ^-^ ^4^ It is documented that IPR is not associated with increased risk of caries or periodontal disease.^4,5^ However, possible contraindications for IPR include severe crowding, poor oral hygiene, or hypersensitivity to temperature variations.^6^ ^,^ ^7^

Before IPR procedure, model analysis is required, because excessive interproximal enamel reduction may cause unfavorable consequences. Among them are dentin hypersensitivity and irreversible enamel furrows that could be a predisposing factor for plaque accumulation.^8^ ^,^ ^9^ Some stripping guidelines suggest that up to 50% of the proximal enamel thickness can be safely removed without compromising dental and periodontal health.^10^ Enamel removal from 0.2 mm to 0.3 mm per proximal surface for the anterior teeth has been described as being safe in the literature.^11^ ^-^ ^15^

Proximal enamel thickness of mandibular incisors is directly related to the amount of IPR that can be safely accomplished without iatrogenic complications. The enamel thickness of mandibular incisors has been previously measured in a few studies through non-destructive and destructive methods. Destructive techniques provide accurate enamel thickness measurement, but they present disadvantages that include orientation problems and unavoidable loss of tooth material.^12^ ^,^ ^16^ ^-^ ^19^ On the other hand, non-destructive techniques, such as radiographs, ultrasound, terahertz imaging, and computed tomography (CT) scanning have been implemented to measure the enamel thickness.^21^ ^-^ ^25^ However, due to their shortcomings in providing accurate measurements,^21^ ^,^ ^25^ ^,^ ^26^ there is a need to consider a technique that can provide more accurate measurements of enamel thickness. Micro CT technology has been introduced to the dental research field as one such method because of its high-resolution results.^20^ ^,^ ^26^ ^-^ ^28^

The purpose of this study was to employ micro-CT scans to measure enamel thickness of the proximal areas of the mandibular incisors. Additionally, this study was designed to compare the enamel thickness between mesial and distal surfaces of the lateral incisors, and to determine the relationship between tooth width and the mean enamel thickness. 

## MATERIAL AND METHODS

Forty-one extracted single-rooted permanent mandibular incisors were collected from private practices in Brazil. No information was available with regard to age, sex, or race of the sample. The teeth were visually selected by one investigator based on the absence of extensive interproximal wear, decay, attrition, abrasion, abfraction, or fracture. The same investigator sorted the teeth into centrals, laterals, right and left incisors, by their anatomical characteristics. The teeth were divided into three groups: Group 1- central incisors (n = 18); Group 2- right lateral incisors (n = 10); and Group 3- left lateral incisors (n = 13). 

### MICRO-CT SCANS

To be scanned by a micro-CT scanner, the roots of the teeth were manually sectioned and separated from the crowns without distorting the enamel margins in the cervical area. The crowns, which initially were placed in 2% agarose suspension, were positioned vertically in a 20-mm diameter SCANCO sample vial, with the long axis parallel to the long axis of the vial, as visually evaluated. 

All scans were taken at the micro-CT facility at a medical school associated with a local university. A SCANCO µ40 scan (µCT 40; Scanco Medical, Switzerland) was used because it produces comparable results with other microtomographic systems.^28^ Scan settings were: 114 µA, 70 kVp, and 300 ms integration time at 10 µm high resolution with isometric voxels of 10 µm^3^ in size. All scans were created with 2048 x 2048 pixels per slice, and the thickness was kept constant at 0.01 mm. 

### AREAS OF MEASUREMENT

####  Mesial and distal surfaces 

**Figure 1:**
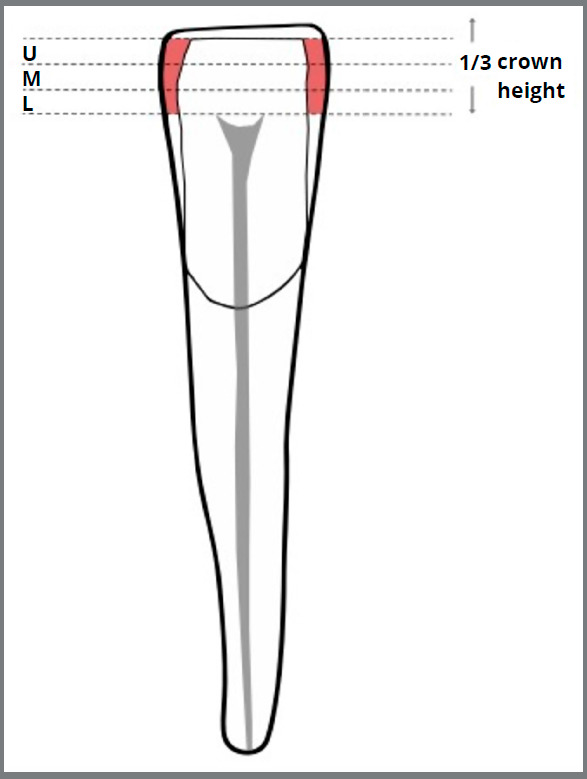
Highlighted areas of measurement on the mesial and distal surfaces on the incisal third of the crown of mandibular incisors: U= upper zone, M= middle zone, L= lower zone.

The vertical position of the contact area represents a starting point and a guide for the initiation of the IPR technique. For the mandibular incisors, the mesial and distal proximal contact areas are located on the incisal third of the crown height.^17^ ^,^ ^30^ For this reason, a fixed area was chosen on the incisal third of the crown to include both the contact area and the widest mesiodistal slice of the tooth. This fixed area was further divided into three zones: lower zone, middle zone, and upper zone (Fig 1). The enamel thickness was measured on the mesial and distal surfaces starting from the most gingival slice of the lower zone and at each slice up to the most incisal slice at the dentinoenamel junction of each tooth (Fig 1). The slices that measured the enamel superior to the incisal level of the dentinoenamel junction were excluded from the measurements.

####  Width 

The maximum interproximal width was measured using a sharpened digital caliper (Mitutoyo, Hampshire, UK). Various studies have demonstrated that the digital caliper is the “gold standard” for tooth-width measurements due to its accuracy, reliability, and reproducibility.^31^

### SOFTWARE ANALYSIS

The sample was scanned with a SCANCO micro-CT. Subsequent image processing was done with SCANCO micro-CT onboard analysis software, in order to minimize post-processing changes of the original data due to data transition. The DICOM images were uploaded to the software, and a manual segmentation was performed to isolate each tooth. Then the orientation of each tooth was checked and, if needed, re-oriented for the tooth’s y-axis to coincide with the anatomical long axis. A threshold of 530 grayscale units was visually selected and applied to differentiate the enamel region. Initially, the number of the scanned slices that represented the total crown height was calculated. This number was divided by three, and thus the number of the slices that represented the incisal third of the tooth was identified. From this number, the number of slices that represented the incisal enamel above the level of the dentinoenamel junction was removed. Manual contouring was completed every ten slices for the rest of the incisal third, followed by the application of a morphing algorithm that was automated to contour the total 100 slices. Running the SCANCO analysis algorithm produced the mean enamel thickness and standard deviation measurements of the highlighted enamel for every 100 slices, each slice at 0.1-mm intervals using the Hildebrand and Rüegsegger^31^ method. Finally, three enamel thickness measurements were found per proximal surface for the three subdivided different zones: upper, middle and lower.

### MASK CONTOURING

**Figure 2:**
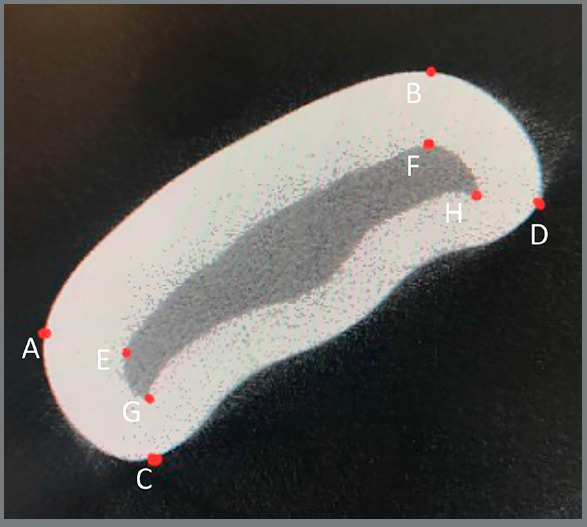
Enamel landmarks in slice in middle zone: A) The outermost convex point of the mesial marginal ridge, B) the distal marginal ridge, C) the junction between the labial and mesial surface, and D) the junction between the labial and distal surface. Dentinal landmarks: E) the dentinal protuberance that corresponds to point A, F) point B, G) point C, and H) point D.

**Figure 3:**
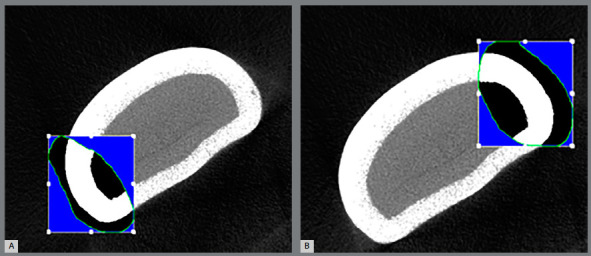
Mask contouring to measure enamel thickness at the proximal areas: A) mesial, B) distal.

Regions of interest were selected in a consistent pattern. A mask was drawn to isolate the region of interest at each slice, with the objective of measuring the enamel thickness. Eight identification landmarks were placed on these regions: four enamel and four corresponding dentinal landmarks (Figs 2 and 3). The mask of interest was achieved using a free-hand drawing tool, and auto-interpolation between the different regions of interest levels produced the total volume of interest for all frames selected. The enamel landmarks were connected with their corresponding dentinal landmarks, to draw the mask for the area of interest (Fig 3).

## BLINDING AND ERROR OF THE METHOD

All measurements were performed by a single-blinded investigator. The intra-rater reliability was assessed by intraclass correlation coefficient (ICCs) and 10% randomly chosen teeth had all micro-CT scans re-measured after an interval of four weeks.

## STATISTICAL ANALYSIS

The statistical analyses were performed using the statistical SPSS software (version 24.0; SPSS, Chicago). Intraobserver random error was estimated using ICCs. Means and standard deviations of proximal enamel thickness were calculated. Data on the mean enamel thicknesses of proximal surfaces of contralateral lateral incisors, of the overall enamel thickness of the contralateral lateral incisors, and of the overall mean enamel thickness between the central and lateral incisors was compared by a Welch two-sided two sample *t*-test. Paired *t*-tests were done on the thicknesses at the lower, middle, and upper zones, with Holm multiple comparison *p*-value adjustments. Spearman’s rank correlation coefficient was used to investigate for possible correlation between the enamel thickness and the tooth width. Statistical significance level of all tests was established at *p*<0.05.

## RESULTS

ICC, ranging from 0.974 to 0.977, was consistently high and showed excellent reproducibility.

Table 1 describes the mean enamel thickness on the mesial and distal surfaces of the mandibular lateral right and left incisors, the mean width and the overall thickness for all the teeth. The mean overall thickness was obtained for both central and lateral incisors by summing the thickness of enamel located on the mesial and distal surfaces of these teeth. 

**Table 1:** Mean enamel thickness on the mesial and distal surfaces, width, and the overall thickness with standard deviations (in mm).

Groups	Width Mean (SD)	Mesial surface Mean (SD)	Distal surface Mean (SD)	Overall Mean (SD)
Central incisor	5.236 (0.322)	-	-	0.56 (0.08)
Right lateral incisor	5.824 (0.39)	0.59 (0.07)	0.55 (0.1)	-
Left lateral incisor	5.705 (0.437)	0.57 (0.1)	0.58 (0.06)	-
Lateral overall	-	0.58 (0.09)	0.56 (0.08)	0.57 (0.07)

*SD= standard deviation. Overall= thickness of enamel located on the mesial and distal surface of incisors.

**Figure 4:**
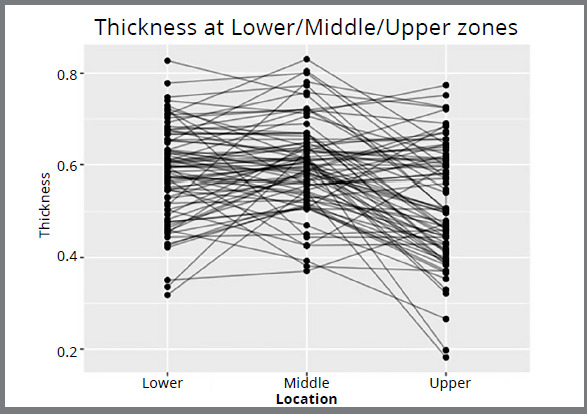
Mean enamel thickness measured for each tooth in correlation with the assessment zone on the incisal third of the crown (upper, middle, and lower).

Table 2 describes the mean enamel thickness measured for each tooth in relation to the assessment zone on the incisal third of the crown (upper, middle, and lower). Figure 4 graphically exhibits data that contributed to establishing the mean thicknesses shown in Table 2.

**Table 2:** Mean enamel thickness per assessment zone (upper, middle, and lower of the incisal third of the crown) and standard deviations (in mm).

Groups	Mean enamel thickness		
	Lower	Middle	Upper
Central incisor	0.569 (0.075)	0.592 (0.08)	0.522 (0.114)
Lateral incisor	0.599 (0.082)	0.603 (0.088)	0.509 (0.089)
Right lateral incisor	0.606 (0.075)	0.596 (0.084)	0.5 (0.108)
Left lateral incisor	0.594 (0.09)	0.608 (0.095)	0.516 (0.075)
Overall	0.586 (0.08)	0.598 (0.084)	0.515 (0.1)

*SD= standard deviation. Overall= thickness of enamel located on the mesial and distal surfaces of incisors.

Table 3 shows that there were no statistically significant differences in the enamel thicknesses between mesial and distal surfaces of lateral incisors (*p*=0.5541) and between the contralateral mesial surfaces (*p*=0.5732) and the distal surfaces (*p*= 0.4197) of the lateral incisors. Consequently, no significant difference was noticed for the overall enamel thickness between the right and left lateral incisors (*p*= 0.8583). In addition, no significant differences were found in the overall enamel thickness between the lateral and central incisors (*p*=0.6931).

**Table 3:** Paired *t*-tests (one-tailed) for mean enamel thickness of various zones: *p*-value, value differences, and range of values (in mm).

Areas of comparison mean enamel thickness	P-value	Difference	Low	High
Overall Mesial-Overall Distal for Lateral incisor	0.5541	0.0131	-0.032	0.058
Mesial Left-Right for Lateral incisor	0.5732	- 0.0204	-0.0943	0.0536
Distal Left -Right for Lateral incisor	0.4197	0.0310	-0.0489	0.1109
Overall Lateral Left-Right	0.8583	0.0053	-0.0563	0.0670
Overall Lateral-Central	0.6931	-0.0091	-0.0558	0.0375
Upper zone-Middle zone	0.0000*	-0.0835	-0.1092	-0.0577
Upper zone-Lower zone	0.0000*	-0.0711	-0.1039	-0.0382
Middle zone-Lower zone	0.2128	0.0124	-0.0072	0.0320

*Significant differences by *t*-test at *p*<0.05.

**Figure 5:**
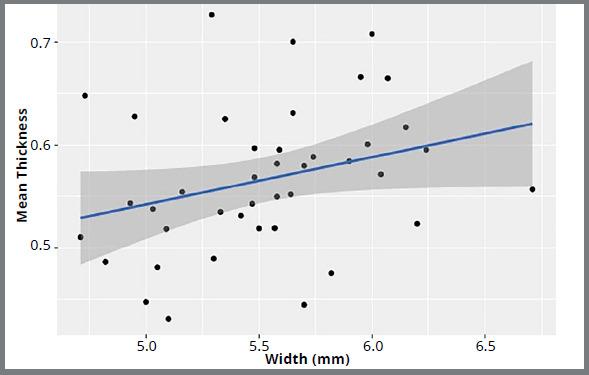
Mean enamel thickness for each tooth, in correlation with tooth width.

The mean thickness of the width of the tooth was modeled, and a line of best fit was found to be Thickness= 0.313 ± 0.4578 Width with R^2^= 0.06855. The slope of Width was found to be different from 0 (*p*= 0.0624). This means that enamel thickness of the mandibular incisor was not significantly correlated with the tooth width. Figure 5 represents mean enamel thickness for all the teeth, correlated with the tooth width.

When the incisal third of the crown was evaluated, the mean enamel thickness at the upper zone was found to be significantly thinner than the mean enamel thickness at the middle and lower zones (Table 3). The lower thickness was not found to differ from the middle thickness (*p* = 0.21), but the upper thickness was found to be different from the lower thickness (*p*= 9.5x10^-5^) and the middle thickness (*p*= 2.3x10^-8^). Confidence intervals for the difference in mean thickness were found to be 0.038-0.104 for the lower zone compared to upper zone enamel thickness, and 0.0577-0.109 for the middle zone compared to upper zone enamel thickness. 

## DISCUSSION

The evaluation of enamel thickness of mandibular incisors has been described in the scientific literature.^12^ ^,^ ^16^ ^-^ ^19^ However, the techniques that have been used for the quantification of the amount of enamel had shortcomings.^21^ ^,^ ^25^ ^,^ ^26^ Therefore, the current study was designed to investigate enamel thickness with the use of micro-CT scans to measure dental enamel thickness due to its high accuracy and reliability of the measurements in dental research.^20^ ^,^ ^26^ ^,^ ^27^ ^,^ ^35^

Measurements in this study were performed at the incisal third of the tooth crown, where anatomically the contact point for the mandibular incisors is located. The mean enamel thickness for each tooth at each slice with 0.1-mm intervals, the mean enamel thickness for all teeth at each slice with 0.1-mm intervals, and the overall mean enamel thickness for proximal surfaces were reported.

The enamel formation of the permanent teeth up to the first molar is completed between the ages of 3.0 to 3.3 years, consequently the lack of information about the age of the sample did not affect the enamel thickness measurements.^32^ Moreover, combining the genders in the study sample agreed with past studies about the lack of sexual dimorphism in proximal enamel thickness.^19^ ^,^ ^33^ ^,^ ^34^

No significant difference was found for the mean enamel thicknesses on the mesial and distal surfaces of mandibular incisors. Similarly, Sarig et al.^17^ reported that the mesial and distal enamel thickness of mandibular incisors were similar. On the other hand, Vellini-Ferreira et al.^16^ found distal enamel significantly thicker than mesial. However, the former study was performed by sectioning the teeth, and therefore, the different results may be attributed to limitations in specimen orientation and loss of tooth material. Furthermore, in the current study no significant difference was found in the overall enamel thicknesses between the right and the left lateral incisors. This outcome is consistent with that found in larger samples of mandibular incisors, such as the Vellini-Ferreira et al.^16^ study that found no difference for the enamel thickness between right and left mandibular incisors.

In this study, no significant difference was found between the mean enamel thicknesses of the central and lateral incisors. This may suggest that it would be clinically safe to apply the same IPR technique and remove equal amounts of enamel from all mandibular incisors. Additionally, no significant correlation was found between tooth width and mean enamel thicknesses of the proximal surfaces. These results indicate that wider incisors do not necessarily have more enamel than narrower incisors, and the size difference can be attributed to larger amount of dentin. Subsequently, tooth size cannot be clinically used to evaluate the amount of enamel reduction. Some authors came to the same conclusion as in this study, and found that the enamel thickness of the teeth was not related to the tooth size.^18^ ^,^ ^36^ Hall et al.^19^ reported a significant correlation between enamel thickness and tooth width. However, conventional radiographs were used, so the results should be viewed with caution.^21^

The lower zone mean enamel thickness was found not to differ significantly from the middle zone mean thickness, whereas, the upper zone mean thickness was found to be significantly different in mean thickness from the lower and middle zones. The upper zone enamel layer was found to be the thinnest for all the teeth, followed by the middle zone enamel thickness and finally the lower zone enamel thickness. This result suggests that the orthodontist should pay attention when approaching the incisal edge during the IPR.

## CLINICAL IMPLICATIONS

There are several reported guidelines for the optimal enamel reduction. Some studies have suggested that a maximum 50% of the enamel thickness can be safely removed without iatrogenic effects.^10^ Other authors recommended that the amount of enamel to be removed during interproximal stripping should vary from 0.2 to 0.3 mm per proximal surface.^11^ ^-^ ^15^ The results of this study suggest that the upper zone mean enamel thickness (0.52 ± 0.10 mm), the thinnest enamel layer, should be used as a guide for determining the maximum amount of enamel that can be safely removed. Thus, it is possible to suggest 0.25 mm as a critical limit of enamel reduction per proximal contact point for the mandibular incisors. The removal of 0.25 mm is effective and safe, and it corresponds to less than 50% of the enamel thickness. However, the orthodontist should not disregard the deviations in proximal enamel thickness between teeth with different morphologies, especially triangular-shaped incisors, and one should customize the enamel reduction per proximal contact point. 

Knowledge of the interdental enamel thickness is critical to deciding the amount of enamel that can be safely removed by professionals during enamel stripping on the proximal surfaces. This study has provided accurate measurements and comparisons of enamel thickness to guide the clinician with confidence when proceeding with IPR.

One limitation of this study is that the sample consisted of teeth from unidentified patients, therefore variables such as gender and ethnicity could not be considered. Despite this limitation, these findings will raise awareness of the amount of IPR that can safely be performed without causing harm.

## CONCLUSIONS

1. There were no significant differences between mean enamel thickness on the mesial and distal surfaces of mandibular incisors.
2. The thinnest enamel of a lower incisor tends to be in the upper portion of the incisal one-third of the tooth.
3. There is no significant correlation between tooth width and mean enamel thickness.
